# Pituitary abscess: a case report and systematic review of 488 cases

**DOI:** 10.1186/s13023-023-02788-1

**Published:** 2023-06-26

**Authors:** Felicity Stringer, Yi Chao Foong, Alanna Tan, Sarah Hayman, Jeffrey D. Zajac, Mathis Grossmann, Justin Ng Yau Zane, Jasmine Zhu, Sujith Ayyappan

**Affiliations:** 1 Barwon Health, Geelong, VIC Australia; 2 St Vincent’s Health, Melbourne, VIC Australia; 3 Monash Health, Melbourne, VIC Australia; 4 Alfred Health, Melbourne, VIC Australia; 5 Eastern Health, Melbourne, VIC Australia; 6 Austin Health, Melbourne, VIC Australia; 7 Monash University, Melbourne, VIC Australia; 8 Royal Hobart Hospital, Hobart, TAS Australia

**Keywords:** Pituitary, Abscess, Hypopituitarism, Rathke’s cleft cyst, Systematic review

## Abstract

**Background:**

Pituitary abscess (PA) is a rare condition and not well understood. We aimed to describe a case and perform a comprehensive systematic review to explore presenting symptoms, radiological findings, endocrine abnormalities and mortality.

**Aim:**

To identify presenting symptoms, radiological findings, endocrinological abnormalities and predictors of mortality for PA.

**Methods:**

We systematically reviewed the literature to identify all case reports of PA. Data regarding presentation, mortality, radiological findings, endocrinological abnormalities and treatment was extracted.

**Results:**

We identified 488 patients from 218 articles meeting the inclusion criteria. Mortality was 5.1%, with days to presentation (OR 1.0005, 95% CI 1.0001–1.0008, *p* < 0.01) being the only identified independent predictor of mortality. Mortality rates have decreased over time, with cases published prior to 2000 having higher mortality rates (OR 6.92, 95% CI 2.80–17.90, *p* < 0.001). The most common symptom was headache (76.2%), followed by visual field defects (47.3%). Classical signs of infection were only present in 43%. The most common imaging feature on magnetic resonance imaging (MRI) was high T2 and low T1 signal of the pituitary gland with peripheral contrast enhancement. Over half (54.8%) were culture negative, with the most common bacterial organism being staphylococcus aureus (7.8%) and fungal organism being aspergillus (8.8%). The most common endocrine abnormality was hypopituitarism (41.1%), followed by diabetes insipidus (24.8%). Whilst symptoms resolved in most patients, persistent endocrine abnormalities were present in over half of patients (61.0%).

**Conclusion:**

PA is associated with significant mortality, with delayed presentation increasing risk of mortality. Ongoing endocrinological abnormalities are common. Given the non-specific clinical presentation, the appearance of high T2, low T1 and peripheral contrast enhancement of the pituitary on MRI should prompt consideration of this rare disease.

## Introduction

Pituitary abscess is a rare but life-threatening condition that is under recognised amongst physicians. Abscesses constitute approximately 1% of diagnosed pituitary lesions [1–5]. The non-specific nature of symptoms and radiological findings contribute to the difficulty in diagnosis, and for this reason it is usually made intra-operatively [1].

Pituitary abscesses are classified as either primary or secondary. Primary pituitary abscesses arise in underlying normal pituitary tissue, while secondary abscesses occurring in pre-existing pituitary lesions. Such lesions include craniopharyngiomas, Rathke’s cleft cysts and adenomas [2, 3].

Several case series of pituitary abscesses have been published over the last few decades. These aimed to better categorise imaging findings [2, 6], presenting symptoms [2, 4, 5] and risk factors [7]. However, radiological findings, symptoms and sequelae reported are heterogenous. Since the most recent systematic review by Agyei et al. in 2017 which included 200 pituitary case reports [1] there have been an additional 288 cases reported, providing a substantial amount of additional clinical data on this rare condition. Therefore, an updated systematic review is timely. We also, in contrast to Agyei et al., have included radiological findings and analysed predictors of mortality which may enable early diagnosis and identify patients at risk of poor outcomes.

Here we present a case report of a pituitary abscess arising in a Rathke’s cleft cyst and a systematic review of the literature of 488 cases. We aimed to explore the presenting symptoms, microbiological profile, radiological findings, and endocrinological sequelae of pituitary abscess. We also aimed to identify risk factors for mortality in this condition.

## Case report

A 25-year-old Australian female presented to the emergency department with 2 days of severe bifrontal, constant headache associated with nausea and rigors. This was on the background of a 3-month history of progressively worsening left-sided frontal headache. These had no association with her menstrual cycle, which remained regular.

She had visited her general practitioner 6 weeks prior, who diagnosed her with migraines. Outpatient non-contrast magnetic resonance imaging (MRI) of her brain was performed 4 weeks prior to admission, and this demonstrated a 19 × 11x13mm pituitary cystic mass, reported as most consistent with a macroadenoma.

She had no significant past medical history, and worked in the hospital as a junior medical officer.

On admission she was alert and orientated, afebrile and with normal vital signs. Neurological exam was unremarkable with no visual field defect or cranial nerve palsies. Her laboratory tests on admission showed a mild neutrophilia (white cell count 11.0 × 10^9^/L, neutrophils 8.9 × 10^9^/L), mildly low T3 (2.0 pmol/L, reference range 3.5–6.5 pmol/L) and T4 (8.7 pmol/L, reference range 9–25 pmol/L) with normal TSH, and other endocrinological investigations within normal range (Table 1).

**Table 1:** Investigation results pre and post-operative, with reference ranges. Abnormal results are shown in bold

Investigations	Pre-operative	Post-operative	Reference range
*Blood tests*			
WCC	11.0	7.9	4.0–11.0 × 10^9^/L
Neutrophils	**8.9**	4.2	2.0–8.0 × 10^9^/L
Sodium	138	143	135–145 mmol/L
*Serum Hormones*			
bHCG	< 2	–	< 5 IU/L
FSH	7	–	2–16 IU/L
LH	5	–	0–75 IU/L
Prolactin	163	-	60–620 mIU/L
GH	1.4	–	< 5.0 ug/L
TSH	0.43	0.82	0.40–4.00 mIU/L
T3	**2.0**	**3.2**	3.5–6.5 pmol/L
T4	**8.7**	**7.8**	9.0–25.0 pmol/L
Cortisol	588	** < 11**	110–550 nmol/L

*bHCG* Beta human chorionic gonadotrophin, *FSH* Follicle stimulating hormone, *GH* Growth hormone, *LH* Luteinising hormone, *T3* Triiodothyronine, *T4* Thyroxine; TSH – thyroid stimulating hormone, *WCC* White cell count

**Fig. 1:**
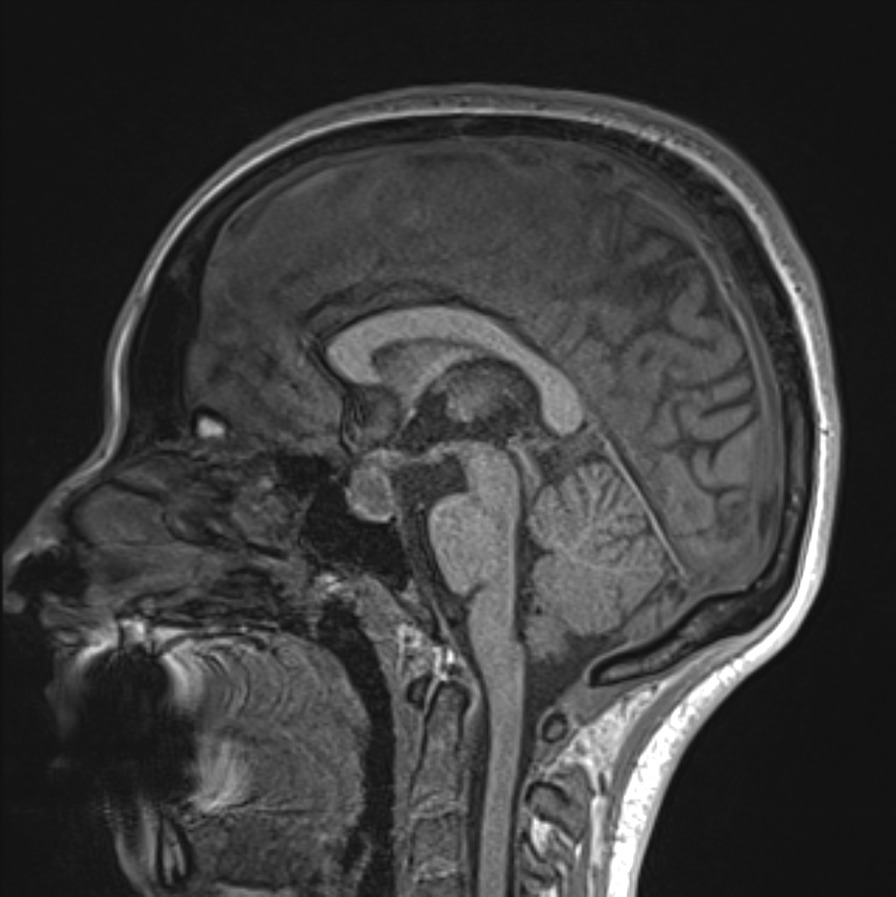
Pre-operative MRI brain scan sagittal T1 sequence showing a hypointense pituitary mass

**Fig. 2:**
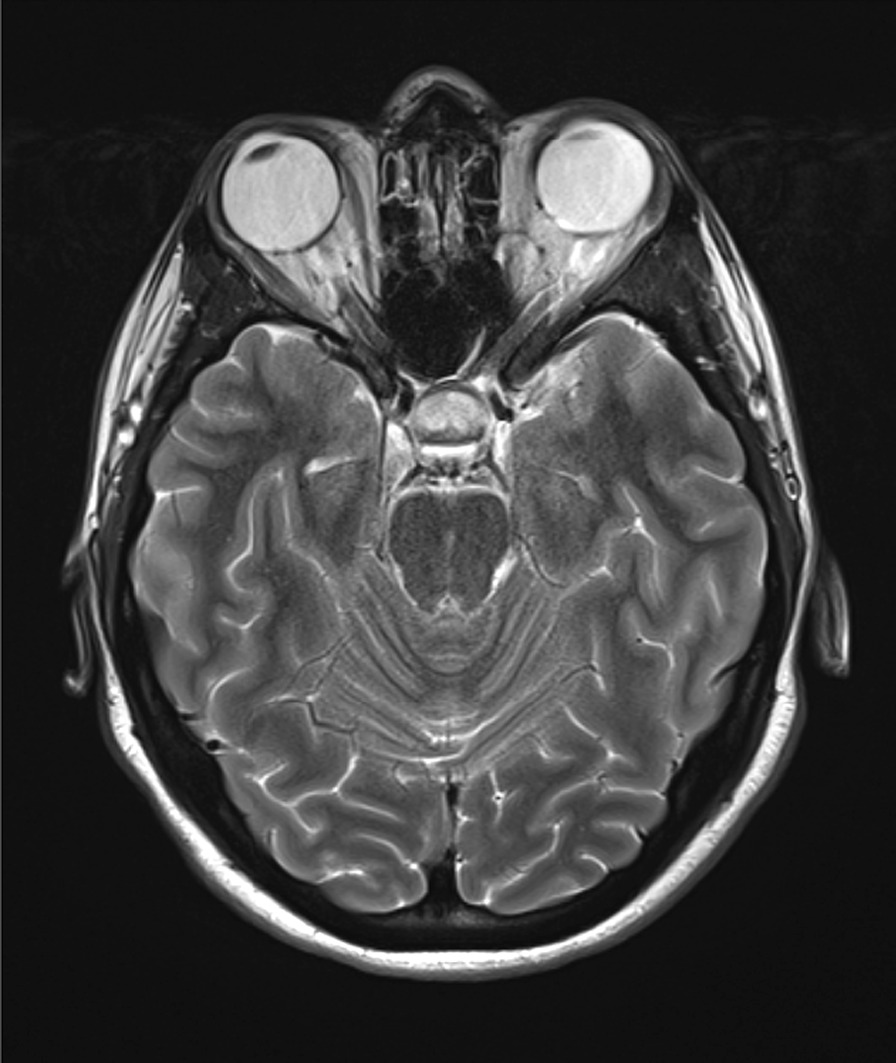
Pre-operative MRI brain scan axial T2 sequence showing a hyperintense pituitary mass

**Fig. 3:**
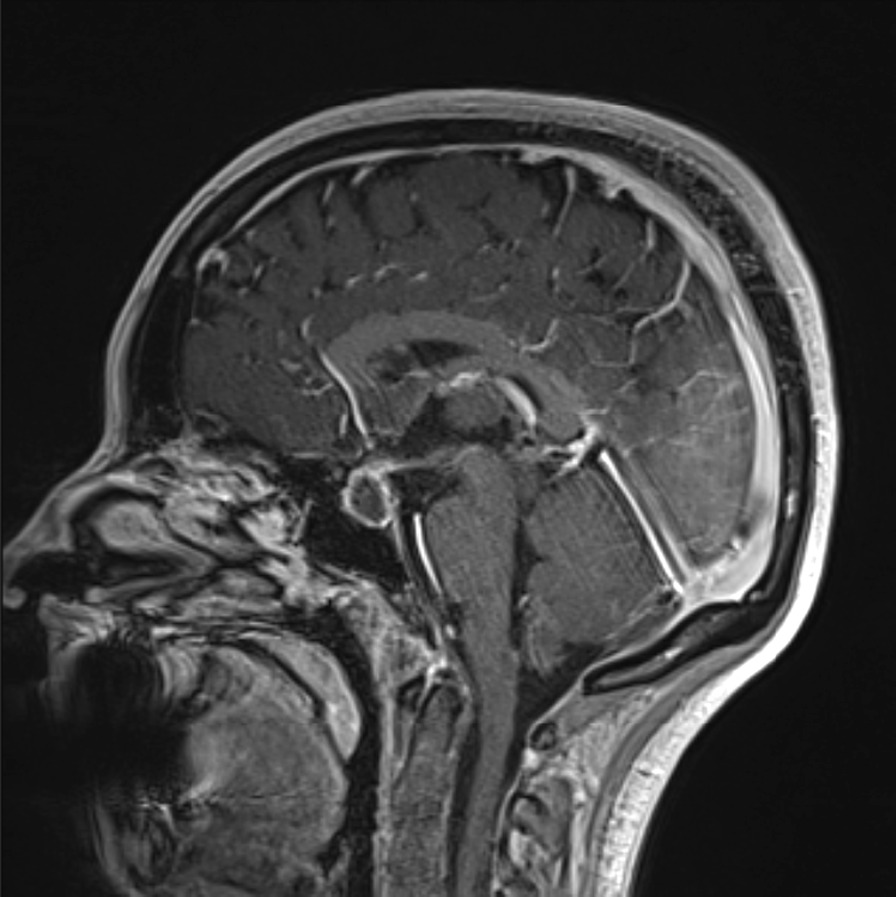
Post-operative MRI brain scan post-gadolinium T1 sequence showing a pituitary mass with peripheral contrast enhancement

An inpatient MRI brain with contrast demonstrated a 10 × 21x13 mm cystic mass occupying the sellar region with suprasellar extension, and peripheral contrast enhancement. The lesion was hypointense on T1 signal and hyperintense on T2 signal (Figs. 1, 2 and 3). The MRI was reported as a cystic pituitary macroadenoma or Rathke’s cleft cyst with a haemorrhagic component.

Immediately after her MRI scan the patient became febrile (peak temperature of 38.6 °C), and developed nuchal rigidity and mild confusion, with difficulty performing serial sevens (where the patient must count down from one hundred by sevens). She was treated empirically for meningo-encephalitis with ceftriaxone 2 g 12 hourly, benzylpenicillin 2.4 g 4 hourly, acyclovir 600 mg 8 hourly and dexamethasone 10 mg 6 hourly intravenously. A lumbar puncture was performed, which showed 1777 × 10^6^ white blood cells, (70% polymorphonuclear and 30% mononuclear cells), protein 0.83 g/L (reference range 0.15–0.45 g/L and glucose 2.3 mmol/L (reference range 2.8–4.2 mmol/L). She was reviewed by the infectious diseases team and the possibility of a pituitary abscess was raised. The patient was transferred to a neurosurgical unit for further evaluation and management.

She underwent trans-sphenoidal drainage the following day, where frank pus was drained, confirming the diagnosis of a pituitary abscess. Histopathology showed features suggestive of a Rathke’s cleft cyst with superimposed infection, but there was no growth on culture.

Post-operatively the patient developed polydipsia and polyuria. Further endocrinological investigations confirmed diabetes insipidus as well as hypocortisolaemia and hypothyroidism, which were managed with desmopressin, cortisone and thyroxine respectively.

She was also treated with a 4-week course of intravenous antibiotics (ceftriaxone and metronidazole), and her symptoms resolved.

The patient now remains on a decreasing dose of desmopressin, cortisone and thyroxine, with regular private endocrinology follow-up to monitor for ongoing medication requirement. Post-operative MRI showed a reduction in size of the peripherally enhancing sellar lesion, measuring approximately 8.5 × 19.8x8mm.

Follow up at 4 weeks post discharge revealed resolution of her headache and meningism with no sign of disease recurrence.

## Method

### Search protocol and inclusion criteria

This systematic review was conducted as per the Preferred Reporting Items for Systematic Reviews and Meta-analyses (PRISMA) statement. [8, 9] In collaboration with an experienced research librarian we devised an overall search strategy and confirmed the search parameters. Articles were identified on research databases PubMed/Medline, Cochrane Trial Register and Embase, as well as 5 articles found through additional searching. The search algorithm was adapted according to each database. Search terms included ‘pituitary diseases’, ‘pituitary gland’, ‘hypopituitarism’, ‘sella turcica’, ‘craniopharyngioma’, ‘pituitary’, ‘sella’, ‘sellar’, ‘intrasellar’, ‘hypophysis’, ‘hypopituitarism’, and ‘Rathke’. These terms were combined with the operator ‘OR’. Each of the words was combined with the word ‘abscess’ by the operator ‘AND’.

Two authors (FS, YF) independently screened the title and abstract of each article to identify potential case reports of pituitary abscesses. Full text articles were then reviewed based on the inclusion criteria to determine whether they were appropriate to be included in this review. Any inconsistencies were discussed and agreement reached.

To be included in the systematic review the articles had to include an original case report of a pituitary abscess and be available in English. Abstracts, poster presentations and review articles that did not include original case reports were not included.

### Data extraction

The following data was extracted from the articles: age and ethnicity of patient, presenting symptoms, signs of infection at presentation, time to presentation, radiographical findings, endocrinological abnormalities at presentation, source of infection, treatment modality, culture growth, duration of antibiotics, outcome including ongoing endocrinological abnormalities, and follow up duration.

## Results

### Study selection

**Fig. 4:**
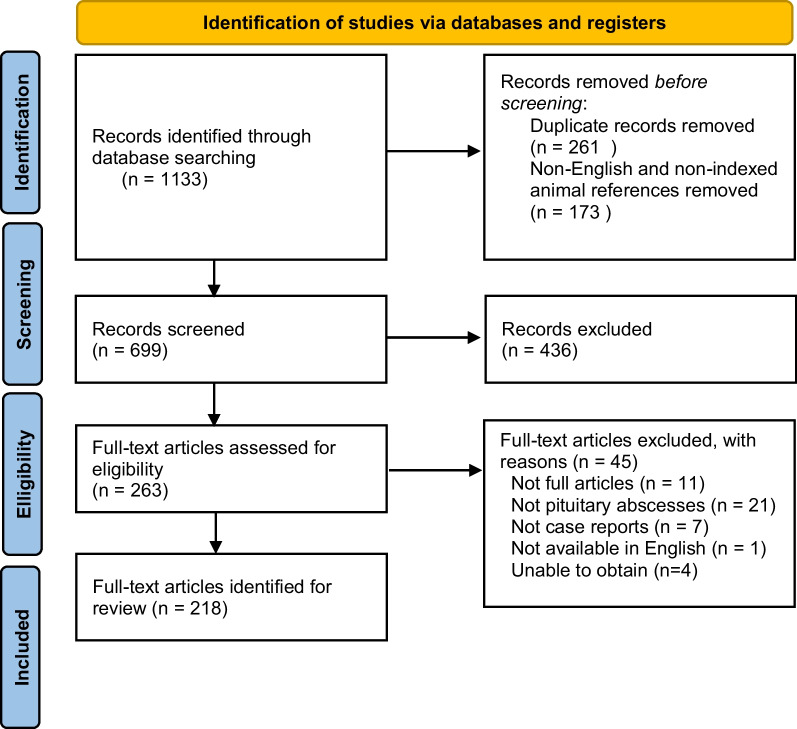
Preferred Reporting Items for Systematic Reviews and Meta-analyses diagram illustrating identification of appropriate articles for systematic review of pituitary abscess

There were 1133 records identified from the initial search protocol. After removal of duplicates and non-English and animal references, a total of 699 articles were retained for further evaluation. A further 477 articles were removed after abstract and full text screening, leaving a total of 218 articles identified for review (Fig. 4). [1–7, 10–220]

### Participant characteristics

We found a total of 218 papers, with a total of 488 patients. Of those, 275 (56.4%) were female, of which 237 (48.6%) were below the age of 60. The most frequent country of origin was China (221) followed by USA (65) and India (35). Mean age was 41.5 years (SD 16.7). There were 25 (5.1%) deaths.

### Symptoms and signs

The most common symptom was headache (76.2%), followed by visual field defects (47.3%), fever (30.7%) and polydipsia and polyuria (27.3%). Menstrual disturbance was a common symptom in 78 out of 237 (32.9%) women of menstrual age (defined as age below 60). Only 210 (43%) had signs of infection, defined as a temperature above 38 degrees celsius or biochemical markers such as raised WCC/CRP and consistent CSF abnormalities (Table 2). Full table of presenting symptoms can be seen in appendix as Table 7.

**Table 2:** Presenting symptoms

Symptoms	N (%)
Headache	372 (76.2%)
Visual field defects	231 (47.3%)
Fever	150 (30.7%)
Polydipsia/polyuria	133 (27.3%)
Vomiting/nausea	96 (19.7%)
Menstrual disturbance	78 (32.9%*)
Weakness	65 (13.3%)

^*^N for this symptom is 237 women of menstrual age defined as age < 60 years. Only values > 5% are included, full table can be seen in appendix as Table 7

Of the 318 patients with sufficiently accurate reporting of time to presentation, median time from onset of symptoms to presentation was 120 days (IQR of 30–360).

### Imaging findings

MRI data was available for 396 patients. The most common radiological features were peripheral contrast enhancement, followed by T2 hyperintensity and T1 hypointensity (Table 3). Full table of imaging findings can be seen in appendix as Table 8. Of those that did not have MRI results available, CT findings were reported in 38. Sellar mass was visible in all cases, with post contrast enhancement in 17 of the 38

**Table 3:** Imaging findings

MRI features	N (%)
Peripheral enhancement	249 (62.9%)
High T2	194 (49.0%)
Low T1	168 (42.4%)
Cystic features	56 (14.1%)
Isointense T1	50 (12.6%)
High T1	37 (9.3%)
Invasion/extension into surrounding structures	35 (8.8%)

Only values > 5% are included, full table available in appendix as Table 8

### Abscess suspected pre-operatively

Whether a pituitary abscess was suspected pre-operatively was adequately reported in 408 cases. Of these, 160 had the correct pre-operative presumed diagnosis (39.2%). Of the remaining 248, the suspected diagnosis was reported in 174. Out of these, the most common diagnosis was an adenoma (27.9%). All other suspected diagnosis were < 5%. See Table 9 in appendix.

### Treatment

The most common treatment modality was transsphenoidal surgery (TSS) in 392 patients (Table 4). Full table of treatment modalities can be seen in appendix as Table 10.

**Table 4:** Treatment modality

TSS	392
Craniotomy	58
Antibiotics only	24
No treatment specified	7
Died prior to treatment	2

Full table can be seen in appendix as Table 9

### Antibiotics pre surgery and culture result

Sufficient pre-operative antibiotic use was reported in 290 patients, of which 156 received pre-operative antibiotics.

Sufficient intra-operative culture results were reported in 431 participants. The most common result was no growth (236, 54.8%). The most common bacterial organism reported was staphylococcus aureus (34, 7.9%) followed by coagulase negative staphylococcus (31, 7.2%). The most common fungal organism was aspergillus (36, 8.4%). Pre-operative antibiotic use was significantly associated with higher odds of having a negative culture result (OR = 1.79, 95% CI 1.06 – 3.06, *p* = 0.02). Full table of culture growth can be seen in appendix as Table 11.

### Endocrinological abnormalities

Results of initial endocrinological testing was reported in 431 patients. Of these, 177 had panhypopituitarism, 107 had diabetes insipidus, and 67 had normal endocrinological testing (15.5%).

Results of immediate post-operative endocrinological testing was reported in 321 patients.

Results of ongoing endocrinological testing was reported in 333 patients. Of these, 92 had persistent panhypopituitarism, 56 had diabetes insipidus, and 128 (38.4%) had normal endocrinological testing (Table 5).

**Table 5:** Endocrinological abnormalities

	Initial (N = 431)	Immediate post-op (N = 321)	Ongoing (N = 333)
Panhypopituitarism*	177	90	92
Low TSH/T3/4	74	72	67
High TSH/T3/4	1	0	0
Low cortisol/ACTH	75	79	65
High cortisol/ACTH	2	0	0
Low GH	16	6	6
High GH	10	1	0
Low FSH/LH/estrogen/testosterone	69	25	26
High FSH/LH/estrogen/testosterone	0	0	0
DI	107	93	56
Normal	69	91	128
High prolactin	10	0	1
Low prolactin	62	12	3
SIADH	1	1	0

^*^Defined as low GH/IGF-1, low cortisol/ACTH, low TSH/T3/T4, low FSH/LH/testosterone/ oestrogen

*ACTH* Adrenocorticotropic hormone, *DI* Diabetes insipidus, *FSH* Follicle stimulating hormone, *GH* Growth hormone, *LH* Luteinising hormone, *SIADH* Syndrome of inappropriate antidiuretic hormone, *T3* Triiodothyronine, *T4* Thyroxine, *TSH* Thyroid stimulating hormone

### Outcomes and follow up

There were 318 cases of primary pituitary abscesses and 147 were secondary pituitary abscesses.

Outcomes were reported in 412 patients. Often full endocrinological panels were not reported, so they were summarised as ongoing endocrinological abnormalities (Table 6).

**Table 6:** Outcomes

Ongoing endocrinological abnormalities	205
Full recovery	126
Ongoing visual deficit	37
Ongoing visual deficit but improved	15 of the 37
Recurrent pituitary abscess	35
Death	25
CSF leak	7
Meningitis	7
Aseptic meningitis	2
Tumour recurrence	2
Ongoing headache	1

Of the 488 patients, there were 25 (5.1%) deaths. Mortality rates decreased over time, with higher mortality rates in cases published prior to 2000 (OR 6.92, 95% CI 2.80–17.90, *p* < 0.001). Backwards stepwise logistic regression was performed. Age, days to presentation, signs of infection, primary or secondary and sex were included in the initial model. Days to presentation (OR 1.0005, 95% CI 1.0001 to 1.0008, *p* < 0.01) was an independent risk factor for increased mortality.

Sufficient follow-up data was available for 391 participants. Median follow up duration was 510 days (IQR 120 – 1461).

## Discussion

### Key findings

- Median time from onset of symptoms to presentation was 120 days (IQR of 30–360)
- Headache and visual field loss are the most common symptoms
- Infective symptoms and biochemical markers of infection are absent in just over half (57%) of cases
- Peripheral rim enhancement post gadolinium, hyperintensity on T2 weighted imaging and hypointensity on T1 weighted images are the most common radiological findings
- 55% of patients have culture negative results, with pre-operative antibiotics associated with higher odds of negative culture
- The mortality rate is 5.1%, with duration of symptoms predicting mortality.
- Endocrinological abnormalities are common (84.5%), and can persist in over half of pituitary abscess patients

This systematic review is the largest to date, and provides key findings to assist clinicians in diagnosis and inform expected prognosis and outcomes. With regards to diagnosis, we have identified the two most common presenting symptoms as being headache and visual field deficits. Just over half of patients will not have classical infective symptoms or signs. An MRI scan with gadolinium is the recommended diagnostic test, and typical findings identified in this review are rim enhancement post-gadolinium injection, combined with hypointensity on T1-weighted imaging, and hyperintensity on T2-weighted imaging. The recommended treatment is surgical drainage via TSS. A high percentage of patients will have negative culture results, with pre-operative antibiotic use increasing the odds of a negative culture. Endocrinological abnormalities are commonly found pre-operatively, and do persist in a high number of patients. There are patients who will have recovery of their pituitary function post-operatively, and thus require ongoing long-term follow up with clinicians. Apart from endocrinological abnormalities the majority of other symptoms completely resolve. The mortality rate is 5.1%, with patient age and duration of symptoms prior to treatment being independent risk factors for mortality.

### Symptoms

The most common symptom is headache (76%), followed by visual field loss. The presence of diabetes insipidus should prompt the clinician to think of more sinister pathologies, as it is present in less than 1% of patients with a pituitary adenoma at initial presentation [221].

There is a low percentage of patients (43%) who present with fever or other infective symptoms or signs. A lumbar puncture was often not performed, or not consistently reported. In our case, the lumbar puncture result was helpful in aiding the diagnosis. Future research in assessing whether lumbar puncture results are consistently abnormal in patients with pituitary abscesses is thus warranted. This prevalence of infective features is consistent with other research [1, 4, 5] and reiterates the importance of not relying on these markers to include or exclude the diagnosis.

### Endocrine abnormalities

Of patients with reported pre-operative endocrinological testing, 84% had abnormal results. The most common abnormality was panhypopituitarism (177, 41%), followed by diabetes insipidus (107, 25%).

There is an improvement in endocrinological abnormalities post operatively, and a further 10% of patients have full recovery of pituitary function on follow-up. A number of these patients can decrease their doses of hormonal supplements, if not cease completely. Diabetes insipidus recovers to a greater degree than other pituitary hormones with time. This has been shown in other studies, with less than a third of patients who required desmopressin in hospital post-operatively requiring it long-term (mean follow up time of 88 months) [4]. This indicates the function of the posterior pituitary recovers to a greater degree than the anterior pituitary post-operatively.

### Radiological findings

Peripheral rim enhancement post gadolinium in combination with hyperintensity on T2 weighted imaging and hypointensity on T1 weighted images were the most common radiological findings in our review, and therefore should be considered typical findings of a pituitary abscess. This is in contrast to pituitary adenomas which are usually isointense or hyperintense on T1 weighted imaging, and have homogenous enhancement post gadolinium [1, 6]. The large case series performed by Gao et al. also found rim enhancement post gadolinium enhancement to be the most common finding in pituitary abscesses, seen in 67% of their patient cohort [2], with Wang et al. finding an even higher rate of 82% [6].

### Primary and secondary pituitary abscesses

Pituitary abscess can be broken up in to primary (those arising from a structurally normal pituitary gland) and secondary (those arising from pre-existing pituitary lesions) [2, 3, 24]. In our analysis, 68% of cases were primary pituitary abscesses, and 32% were secondary. This is consistent with previous reports [1, 54, 81]. Patients with Rathke’s cleft cysts have been shown to have a higher rate of formation of pituitary abscesses post TSS than what has been reported post TSS for other indications [7]. However, in our patient’s case this was her first presentation of a pituitary mass and she had not had any previous pituitary surgery.

### Culture results

55% of patients with culture sent from surgical exploration of their pituitary abscess reported no growth. Our data is the first to show that patients who receive pre-operative antibiotics are at a greater risk of negative culture result. However, given we have found delay to presentation and treatment as an independent risk factor for increased mortality, as well as other differentials often considered pre-operatively (such as meningitis), it is still recommended to give empiric antibiotics.

Staphylococcus aureus (34, 7.8%) and coagulase-negative staphylococcus (31, 7.2%) were the two most common bacteria grown, and empiric antibiotics should cover these two organisms. Aspergillus was the most common organism found responsible (8%), and thus appropriate anti-microbial coverage should also be considered, particularly amongst immunosuppressed individuals.

### Treatment modality

Transsphenoidal surgery (TSS) was the most common treatment modality in the majority of cases (80%). This remains recommended management throughout the literature for both diagnostic and therapeutic reasons, by reducing the mass effect and providing source control of the infection, whilst being less invasive than a craniotomy [1, 5, 54]. Patients who were treated with a craniotomy (12%) had complicated abscesses, were in a centre which did not have access to TSS, or were distant case reports. Craniotomy is a higher risk procedure, with potential exposure of other intracranial structures to the infective process [1]. Patients treated with antibiotics alone (5%) were deemed not medically safe to undergo surgery or refused the procedure. This remains controversial, as surgical drainage and histopathological diagnosis of the infective material is required to confirm the diagnosis of a pituitary abscess [54]. The largest clinical case series to date by Gao et al. [2] excluded patients treated by antibiotics alone due to the lack of post-operative histopathological evidence of a pituitary abscess.

### Outcomes

A key finding from this review is that most symptoms attributable to pituitary abscess do improve post TSS. This is in keeping with findings from surgical recovery post TSS for other indications [222–224] and is due to the physical pathology causing these symptoms being removed. Previous evidence shows all patients without recurrence of pituitary abscess have resolution of headache [1], emphasising that these patients should be closely reassessed for potential recurrence.

Recurrent pituitary abscess was found in 8.5% of patients in this review. This is lower than previous reports of 10–25% [1, 4, 5, 54], indicating that recurrence is decreasing with time. This is likely due to improvements in surgical techniques, as well as antibiotic coverage and duration.

### Mortality

The mortality rate found in this systematic review was 5.1%, with days to presentation being the only independent risk factor identified. The likelihood of mortality with pituitary abscesses is decreasing with time. This is again likely due to improved surgical techniques and increased accessibility of MRI scans, but could be contributed to be reporting bias. This mortality rate is consistent with other reviews, showing a mortality rate of 4.5–8% [1, 5]. Deaths are due to pituitary dysfunction and/or spread of infection and resulting sepsis.

### Limitations

A limitation of this systematic review is the reliance on case reports, with a number of studies having a poor level of quality. The case reports contained varied amounts of clinical information and specific investigation results. It is likely there have been many cases of pituitary abscesses not reported in the literature, leading to publication bias. There may be a reporting bias which have influenced our findings.

There are inconsistencies between different case reports in stating exact timing of endocrinology testing pre and post-operatively, as well as potential differences in laboratory values and reference ranges. There are some studies which report pan-hypopituitarism without giving exact endocrinological data.

## Conclusion

Pituitary abscess has a high mortality rate, with duration of symptoms found to be an independent predictor of mortality. Patients presenting with headaches, visual fields defects and hormonal abnormalities should undergo further MRI scanning with gadolinium. Typical radiological findings of peripheral rim enhancement post gadolinium, hyperintensity on T2 weighted imaging, and hypointensity on T1 weighted imaging should raise suspicions of an abscess. While infective features should raise the index of suspicion, lack of infective features should not be a deterrent. The role of lumbar puncture is unclear at this stage, and future studies reporting on lumbar puncture results will be useful. Management should include urgent hormonal replacement, broad spectrum antibiotics and TSS. Culture results are often negative, and pre-operative antibiotics are a risk factor. Symptoms resolve in the majority of cases, and endocrine abnormalities can improve in just under half of cases.

## Data Availability

The datasets analysed during the current study available from the corresponding author on reasonable request.

## Appendix: 1 A. Search protocol

*PubMed*

(Abscess[mh] OR abscess*[tw]) AND (Pituitary Diseases[mh] OR Pituitary gland[mh] OR Hypopituitarism[mh] OR Sella turcica OR Craniopharyngioma[mh] OR pituitary OR sella OR sellar OR intrasellar OR hypophysis OR Hypopituitarism OR Craniopharyngioma OR Rathke*).

NOT (Animals[mh] NOT humans[mh]).

*Embase*

(exp abscess/ or abscess.mp or abscesses.mp) and (exp hypophysis/ or exp hypopituitarism/ or exp sella turcica/ or exp craniopharyngioma/ or pituitary.mp or hypophysis.mp or hypopituitarism.mp or sella.mp or sellar.mp or intrasellar.mp or suprasellar.mp or craniopharyngioma.mp or rathke.mp or rathke’s.mp).

Limit to Humans.

*Cochrane Trials Register*pituitary in Title Abstract Keyword AND abscess in Title Abstract Keyword

Google Scholar.

Pituitary abscess was searched.

See Tables

**Table 7:** Presenting symptoms

Symptoms	N (%)
Headache	372 (76.2%)
Visual field defects	231 (47.3%)
Fever	150 (30.7%)
Polydipsia/polyuria	133 (27.3%)
Vomiting/Nausea	96 (19.7%)
Menstrual disturbance	78
Weakness	65 (13.3%)
Altered GCS	23 (4.7%)
Fatigue	22 (4.5%)
Meningism	18 (3.7%)
Syncope/presyncope	18 (3.7%)
Anorexia	13 (2.7%)
CN palsies	10 (2.0%)
Hypophrodisia	8 (1.6%)
Neck stiffness	8 (1.6%)
Photophobia	8 (1.6%)
Weight loss	7 (1.4%)
Galactorrhea	7 (1.4%)
CSF rhinorrhoea	6 (1.2%)
Sepsis	4 (0.8%)
Cold intolerance	4 (0.8%)
Dementia	3 (0.6%)
Acromegaly	3 (0.6%)
Seizure	3 (0.6%)
Growth retardation	3 (0.6%)
Impotence/Erectile dysfunction	3 (0.6%)
Ptosis	3 (0.6%)
Psychosis	2 (0.4%)
Proptosis	2 (0.4%)
Weight gain	1 (0.2%)
Photosensitivity	1 (0.2%)
Hemiparesis	1 (0.2%)
Phonophobia	1 (0.2%)
Palpitations	1 (0.2%)
Amnesia	1 (0.2%)
Speech disturbance	1 (0.2%)
Shock	1 (0.2%)
Myalgia	1 (0.2%)
Dry skin	1 (0.2%)
Rash	1 (0.2%)
Facial numbness	1 (0.2%)

*CN* Cranial nerve, *CSF* Cerebrospinal fluid, *GCS* Glasgow Coma Scale

7,

**Table 8:** Imaging findings

MRI features	N (%)
Peripheral enhancement	249 (62.9%)
High T2	194 (49.0%)
Low T1	168 (34.4%)
Cystic features	56 (14.1%)
Isointense T1	50 (12.6%)
High T1	37 (9.3%)
Invasion/extension into surrounding structures	35 (8.8%)
Isointense T2	19 (4.8%)
Mixed T2	11 (2.8%)
Mixed T1	11 (2.8%)
Diffusion restriction	11 (2.8%)
Heterogenous enhancement	7 (1.8%)
Necrosis	5 (1.3%)
Low T2	3 (0.6%)
Haemorrhage	1 (0.3%)
No abnormality	1 (0.3%)

*MRI* Magnetic resonance imaging

8,

**Table 9:** Pre-operative suspected diagnosis (N = 408)

Suspected diagnosis	N (%)
Adenoma	114 (27.9)
Rathke’s cleft cyst	15 (3.7)
Craniopharyngioma	14 (3.4)
Meningitis	9 (2.2)
Hypophysitis	7 (1.7)
Apoplexy	4 (1)
Adenoma with haemorrhagic component	3 (0.7)
Cerebral abscess	2 (0.5)
Stroke	2 (0.5)
Cyst	1 (0.2)
Optic neuropathy	1 (0.2)
Sarcoidosis	1 (0.2)
Subdural empyema	1 (0.2)

9,

**Table 10:** Treatment

TSS	392
Craniotomy	58
Antibiotics only	24
No treatment specified	7
Died prior to treatment	2
Surgery, not specified	2
Surgical exploration only	1
Stereotactic biopsy and drainage	1
Right total ethmoidectomy, maxillary antrostomy, sphenoidotomy	1

*TSS* transsphenoidal surgery

10 and

**Table 11:** Culture growth

No growth	236
Aspergillus	36
Staphylococcus aureus	34
Coagulase negative staphylococcus	31
Of which staph epi	16
Staphylococcus lugdunensis	4
Staphylococcus capitis	3
Staphylococcus haemolyticus	1
Alpha streptococcus	9
Methicillin resistant staphylococcus aureus	9
Tuberculosis	7
Escherichia coli	6
Beta streptococcus	5
Proteus mirabilis	5
Staphylococcus, not specified	5
Strep pneumonia	5
Corynebacterium	4
Gram negative bacteria not specified	4
Pseudomonas aeruginosa	4
Other pseudomonas species	3
Gram positive bacteria not specified	3
Klebsiella pneumonia	3
Klebsiella ozaenae	3
Methicillin resistant staphylococcus epidermidis	3
Mycobacterium not specified	3
Propionibacterium acnes	3
Streptococcus, not specified	3
Actinomycosis	2
Bacteroides	2
Candida albicans	2
Candida glabrata	2
Micrococcus	2
Prevotella melaninogenica	2
Toxoplasmosis	2
Acinetobacter baumanni	1
Acinetobacter iwoffii	1
Acinetobacter not specified	1
Anaerobic streptococcus	1
Brucellosis	1
Citrobacter	1
Enterobacter aerogenes	1
Enterococcus faecium	1
Enterococcus faecalis	1
Fusobacterium	1
Listeria	1
Mycobacterium fortuitum	1
Non haemolytic streptococcus	1
Peptococcus	1
Propionibacterium granulosum	1
Salmonella typhi	1
Staphylococcus not further specified	1
Stenotrophomonas (Xanthomonas) maltophilia	1

^11^.

## Abbreviations

ACTH: Adrenocorticotrophic hormone
bHCG: Beta human chorionic gonadotrophins
CT: Computerised tomography
DI: Diabetes insipidus
FSH: Follicle stimulating hormone
GH: Growth hormone
LH: Luteinising hormone
MRI: Magnetic resonance imaging
PA: Pituitary abscess
SIADH: Syndrome of inappropriate antidiuretic hormone
T3: Triiodothyronine
T4: Thyroxine
TSH: Thyroid stimulating hormone
TSS: Transsphenoidal surgery
WCC: White cell cells
